# Supplementary material for: Practical considerations for noise power spectra estimation for clinical CT scanners
Source: J Appl Clin Med Phys. 2016 May 8;17(3):392–407. doi: 10.1120/jacmp.v17i3.5841 (PMC5690921; doi:10.1120/jacmp.v17i3.5841)
Supplement: Supplementary file 1 — Supplementary Material [file ACM2-17-392-s001.docx]

**Practical considerations for noise power spectra estimation for clinical CT scanners**

**Abstract:** Local noise power spectra (NPS) have been commonly calculated to represent the noise properties of CT imaging systems, but their properties are significantly affected by the utilized calculation schemes. In this study, the effects of varied calculation parameters on the local NPS were analyzed, and practical suggestions were provided regarding the estimation of local NPS for clinical CT scanners. The uniformity module of a Catphan® phantom was scanned with a Philips Brilliance 64 slice CT simulator with varied scanning protocols. Images were reconstructed using FBP and iDose^4^ iterative reconstruction with noise reduction levels 1, 3, and 6. Local NPS were calculated and compared for varied region of interest (ROI) locations and sizes, image background removal methods, and window functions. Additionally, with a predetermined NPS as a ground truth, local NPS calculation accuracy was compared for computer simulated ROIs, varying the aforementioned parameters in addition to ROI number. An analysis of the effects of these varied calculation parameters on the magnitude and shape of the NPS was conducted. The local NPS varied depending on calculation parameters, particularly at low spatial frequencies below ~0.15 mm^-1^. For the simulation study, NPS calculation error decreased exponentially as ROI number increased. For the Catphan® study the NPS magnitude varied as a function of ROI location, which was better observed when using smaller ROI sizes. The image subtraction method for background removal was the most effective at reducing low-frequency background noise, and produced similar results no matter which ROI size or window function was used. The PCA background removal method with a Hann window function produced the closest match to image subtraction, with an average percent difference of 17.5%. Image noise should be analyzed locally by calculating the NPS for small ROI sizes. A minimum ROI size is recommended based on the chosen radial bin size and image pixel dimensions. As the ROI size decreases the NPS becomes more dependent on the choice of background removal method and window function. The image subtraction method is most accurate, but other methods can achieve similar accuracy if certain window functions are applied. All dependencies should be analyzed and taken into account when considering the interpretation of the NPS for task-based image quality assessment.

**Keywords:** Noise Power Spectrum, Computed tomography, Background removal, iterative CT reconstruction, image quality assessment

**I. INTRODUCTION**

The noise power spectrum (NPS), as a more thorough noise descriptor than pixel standard deviation, describes both the magnitude and spatial frequency characteristics of image noise, which plays a critical role in analyzing and optimizing imaging system performance.[^1-5^](#_ENREF_1) It is most often integrated with other metrics to assess image quality for specific tasks,[^6-9^](#_ENREF_6) and has been commonly utilized in the development, characterization, optimization, and comparison of many new imaging technologies such as computed radiography,[^10^](#_ENREF_10) digital mammography,^11-12^ storage phosphors for dental x-ray,[^13^](#_ENREF_13) and other devices in a pre-clinical^14-15^ and clinical^16-17^ environment.

There is, however, a fundamental limitation for the usage of the NPS for CT image noise assessment: the requirement of wide-sense stationary noise.[^18^](#_ENREF_18) In other words, the image mean values must be constant across the entire image and the covariance of the image data must depend only on the relative position between image data points. Due to the intrinsic physics of the CT acquisition process, volumetric CT images violate this condition; this has been reported as a known problem.[^18-20^](#_ENREF_18) NPS calculation methods should therefore consider this limitation and attempt to acquire image data samples with more stationary noise properties by varying experimental conditions. One such approach is to employ the concept of a local NPS,[^4^](#_ENREF_4), [^20-22^](#_ENREF_20) only analyzing the noise in a localized region of interest (ROI) where the noise is approximately wide-sense stationary.

Depending on the chosen calculation parameters, there is a significant amount of variability in the local NPS. First, the frequency-domain sampling of the NPS depends on the selected ROI size and the number of ROIs to be averaged within the images. As the size increases, the spatial frequency resolution of the NPS also increases, but there are fewer ROIs available for averaging, resulting in more statistical fluctuations. NPS accuracy will be improved by increasing frequency resolution and decreasing statistical fluctuations; therefore, both ROI size and number of ROIs have an effect on the accuracy of the NPS. Additionally, if the NPS is used to evaluate quantum noise, excluding non-stochastic image properties, all background trends need to be removed from the image sample prior to NPS calculation. The choice of background removal method has a significant effect on the NPS, particularly in the low spatial frequency domain. Thirdly, prior to Fourier transformation a rectangular window function can be applied to the data to improve signal clarity, and the choice of a particular window function and corresponding parameters affects the resulting calculated NPS.

Although the local NPS is dependent on chosen calculation parameters, many different calculation conditions have been used in previous literature on this subject, with each study only utilizing one fixed set of calculation parameters. Previous studies have utilized various square ROI sizes, with side lengths of 128 pixels,^4, 21-22^ 64 pixels,[^23^](#_ENREF_23) and 32 pixels.[^19^](#_ENREF_19) Similarly, multiple background removal methods have been proposed and implemented. Subtraction of two consecutively acquired image sets is the most common method for background removal^4, 20-21^, but various other background removal methods have also been proposed and implemented, such as the subtraction of a first-order or second-order polynomial fit[^14^](#_ENREF_14), [^22^](#_ENREF_22), [^24^](#_ENREF_24) to the image data. Little has been done in previous literature either to explain the details of background removal methods or to analyze the effect of different background removal methods on the calculated NPS. Additionally, some studies have opted not to apply a window function prior to Fourier transformation,^21-22^ while others have applied Hann and rectangular window functions to the data.[^20^](#_ENREF_20) Moreover, the effect of calculation parameters on the local NPS can vary depending on the CT acquisition and reconstruction parameters. For example, commercial iterative reconstruction algorithms have been developed recently with the purpose of improving CT image quality by reducing the noise magnitude. However, these algorithms may produce reconstructed images with different spatial noise properties, and may further deviate from the condition of wide-sense stationary noise, when compared to conventional filtered back-projection (FBP) reconstruction.^5, 20-21, 25^ Understanding the effect of varied calculation parameters on NPS accuracy is necessary for a comprehensive analysis of image noise properties.

In this paper, we compared the calculated local NPS under varied ROI sizes and locations, for four different image background removal methods and four window functions, using two image reconstruction algorithms (a conventional FBP algorithm and iDose^4^, an iterative algorithm from Philips), via phantom-based and computer-generated imaging studies. The purpose of this study is to understand the effect these dependencies have on the magnitude, shape, accuracy, and smoothness of the NPS, to assess the utility of the local NPS as a noise metric for CT image quality assessment, and to provide practical suggestions for clinical CT scanners.

**II. METHOD AND MATERIALS**

**A. NPS Calculation Formalism**

The noise power spectrum of an image vector **g** can be calculated by applying the discrete Fourier transform: [^18^](#_ENREF_18), [^20^](#_ENREF_20)

. (1)

Here *K* is the covariance matrix of the image data and $\bar{\mathbf{g}}$ represents the expectation (i.e. mean) values of the image data while **g** is a single image acquisition. When **g** represents the entire image, assuming that the noise in **g** is wide-sense stationary, Equation (1) is the calculation of the global NPS. However, CT image noise on a global scale violates this assumption of wide-sense stationary noise; therefore the estimation of the NPS in CT images should be calculated locally. The three-dimensional (3D) local NPS for volumetric CT can be calculated for any ROI **gʹ** within image **g** as below:

. (2)

Here, the NPS is scaled proportionally to the CT voxel dimensions in millimeters (*v_x_, v_y_, v_z_*) and inversely proportional to the number of ROI voxels in each dimension (*N_x_, N_y_, N_z_*) in order to compare spectrums calculated from images with varying voxel dimensions and ROI sizes. A multi-dimensional Fourier transform can be performed on volumetric images to obtain a NPS with the same dimensionality. For a more convenient visual representation of the 3D NPS, the two-dimensional (2D) central transverse slice (i.e. where *f_z_* = 0) is usually extracted, from which a one-dimensional (1D) radial profile is generated. In this study, we computed the 2D central transverse slice via the synthesized slice method,[^24^](#_ENREF_24) in which ensemble averaging is performed on a set of 2D noise power spectrums:

. (3)

Here *N_ROI_* is the number of 2D spectrums utilized for averaging. Pixel dimensions (*p_x_*, *p_y_*) are substituted here for voxel dimensions to denote a 2D process. The factor *Λ_z_* is related to the length over which the spectrums are averaged. Since *Λ_z_* has units of length, the NPS in both Eq. 2 and Eq. 3 will have dimensions of variance multiplied by volume, in this case HU^2^ mm^3^, where HU stands for CT Hounsfield numbers.

For all acquired images in this study, the NPS was calculated via the synthesized slice method. Comparison of the NPS for various calculation parameters (*N_x_*, *N_y_*, *N_ROI_*, and) and local image ROI inputs ($\mathbf{g}'$) was conducted. By utilizing the radial symmetry of the 2D NPS, a 1D spectrum can be generated by binning the data according to its radial spatial frequency, followed by averaging the data in each bin.[^21^](#_ENREF_21), [^24^](#_ENREF_24) The radial bin size for all calculations was selected as 0.025 mm^-1^. This choice was determined by manual tuning to obtain appropriate spectral smoothness, and is the same as that reported in a similar study.[^21^](#_ENREF_21) In practice, the magnitude of the NPS at the spatial frequency origin (*f_x_* = *f_y_* = 0) is difficult to estimate accurately for finite data, as has been reported in previous literature.[^23^](#_ENREF_23) Since the radial, 1D NPS varies linearly at low spatial frequencies,[^4^](#_ENREF_4) the zero-frequency value of the 1D NPS in this study was estimated via linear extrapolation.

**B. Background Noise Removal Methods and Window Functions**

Ideally, the expectation value of the CT number at each unique voxel location in an image set should be determined by averaging a sufficient number (on the order of 10^2^) of repeated scans acquired under identical experimental conditions. However, this is not practically feasible, so it is common practice to estimate the expectation value of each pixel in a ROI, , using a limited amount of acquired images. The result after estimation is the non-stochastic ROI data, which is subsequently subtracted to obtain data containing only quantum noise. In this study, four different background removal methods were utilized to calculate the local NPS; each method is briefly explained and compared here. With the exception of image subtraction, these methods only require one image acquisition to estimate .

**B.1 Image Subtraction-based Method**

In this method, two image sets, which are acquired using identical experimental conditions, are subtracted from each other voxel-by-voxel, removing the non-stochastic image information without direct estimation of expectation values. The result is a residual noise image, with twice the noise variance of an individual sample. As such, when using this method the calculated NPS must be divided by two to account for the doubling of noise magnitude caused by the subtraction process.[^21^](#_ENREF_21), [^24^](#_ENREF_24)

**B.2 First-order Polynomial Fitting Using Residual Sum of Squares**

As opposed to the image subtraction method, which requires two scans, the expectation value for each pixel of a ROI can also be estimated from a single image by calculating the coefficients of a 2D polynomial function. For example, this method was utilized to estimate the background signal of digital X-ray imaging*.*[^26^](#_ENREF_26) In brief, for a ROI with *m* row elements and *n* column elements, the first-order polynomial function fitting for the image background can be defined as:

. (4)

All fitting coefficients *C_i_* (for *i = 1, 2, 3*) are estimated by minimizing the residual sum of squares (RSS), where the RSS is defined as:

. (5)

Here, the weighting factor *w_mn_* was chosen as the inverse square of the ROI pixel intensity, . This method will be designated as RSS first-order.

**B.3 Second-order Polynomial Fitting Using Residual Sum of Squares**

Similar to the first-order polynomial fitting, the second-order cases can also be utilized to estimate for a ROI, designated as RSS second-order.[^26^](#_ENREF_26) The aforementioned method can be extended to a second-order polynomial fitting, with the function being defined as:

. (6)

All fitting coefficients *C_i_* (*i = 1 to 6*) are estimated in the same way as that described in Section II.B.2, by minimizing the RSS second-order function for each coefficient.

**B.4 First-order Fitting Using PCA**

Principal components analysis (PCA) can also be used for first-order surface fitting, and in some instances the results improve upon least squares estimation methods.[^27^](#_ENREF_27) Surface fitting is performed via orthogonal regression, minimizing the perpendicular distances between the data and a first-order planar fit.[^28^](#_ENREF_28) For 3D data (e.g. two dimensions for the image pixel positions and one for the pixel HU value), the first two principal components of the data are two vectors which span the best-fit plane while the third component is orthogonal to the first two and corresponds to the normal of that plane. The best-fit plane with this normal passes through the point which is the mean of each variable. For this study all 2D image ROIs were arranged as a set of 3D points (pixel row, pixel column, and pixel HU value) to perform the fitting process. PCA was then used to analyze the three principal components of this data, which describe the best fit plane.

**B.5 Window Function Application**

Prior to calculating the Fourier transform, but after the background removal method has been performed, a rectangular window function can be applied to the ROI to improve signal clarity. Four window functions which are commonly utilized prior to finite DFT, as determined by literature review,[^29^](#_ENREF_29) were applied in this study: the Hann (or Hanning) function, the Hamming function, the flat top window function, and the four-term Blackman-Harris function.

**C. Data Acquisition & ROI Selection**

**C.1 Catphan® Phantom**

The uniform module section of the Catphan® 504 phantom (The Phantom Laboratory, New York, USA), CTP486, was scanned to obtain uniform CT images for NPS analysis. The CTP486 module is cast from a uniform material that has a CT number within 2% (0-20 HU) of water with a diameter of 150 mm. Figure 1 shows the Catphan® phantom and a CT slice acquired of the uniformity module.

**C.2 Computational Simulation for NPS Calculation Accuracy Verification**

NPS calculation accuracy cannot be appropriately assessed for acquired data due to the absence of a ground truth. Therefore, in order to verify NPS calculation accuracy for different ROI sizes, ROI number, background removal methods, and window functions, simulated ROIs with a predetermined NPS were generated as a ground truth by inverting the process described in Eq. 3. An image of the Catphan® phantom acquired with the scanning parameters of 120 kVp and 250 effective mAs, and reconstructed by the FBP algorithm using the C filter, was used to act as a reference image. This image set was chosen as it contains the most noise of all the images acquired in this study. First, the 1D NPS was calculated from this reference image using a 128-pixel ROI located in the image center and the image subtraction background removal method, with no window function applied, and was used as a reference, ground truth NPS profile. The 128-pixel ROI and the image subtraction background removal method used to compute this reference 1D NPS are the most commonly used calculation parameters as reported in other previous literature.^4, 20-21, 30^ Five 2D NPS with radial symmetry, one for each ROI size used in this study (128, 96, 64, 48, 32), were generated from the reference 1D NPS profile using interpolation. In order to analyze the NPS calculation accuracy for an extensive number of ROIs, the inverse Fourier transform of each 2D NPS profile was then calculated 600 times, maintaining a constant magnitude but introducing random complex phase shifts into each individual calculation, to generate 600 randomly noisy ROIs ($\mathbf{g}'$-) for each size. Without loss of generality, we chose a uniform background trend = 20 HU, and added it to each generated noise ROI to obtain$\mathbf{g}'$. This value for the uniform background trend was chosen by calculating the average ROI value from the uniformity module of the Catphan® phantom. Finally, ROIs were scaled according to the appropriate pixel dimensions (p_x_ & p_y_) and ROI sizes (N_x_ & N_y_). The parameters p_x_ and p_y_ were set to 0.68 mm, approximately equal to the pixel dimensions of the reference image.

For each set of 600 simulated ROIs, 300 2D NPS were calculated using Eq. 3 and the four different background removal methods stated in Section II.B. Note that for the image subtraction removal method all 600 ROIs must be utilized to calculate 300 individual spectra, while the other three methods only require the first 300 ROIs. To analyze the effect of ROI number on NPS accuracy, the ensemble averaged NPS of *N_ROI_* spectrums under the same calculation condition was obtained for *N_ROI_* = 1, …, 300. For each ensemble averaged NPS a 1D radial NPS was calculated as described in Section II.A; thus for each combination of ROI size, background removal method, and window function, 300 1D spectrums were obtained. These results were all compared to the reference one-dimensional NPS profile to assess the accuracy of different calculation parameters (ROI size, ROI number, background removal method, and window function).

**C.3 Data Acquisition**

CT scans of the Catphan® phantom were acquired with a Philips Brilliance 64 slice CT simulator (Philips Healthcare, Cleveland, OH). All CT acquisition and reconstruction parameters were selected to mimic routine clinical protocols at our institution. The phantom was scanned with two tube potential settings (120 and 140 kVp) and two fixed tube current values (250 and 500 effective mAs). Other scan parameters included: 64 x 0.625 mm collimator setting, 0.5 second rotation time, pitch 0.638, and standard resolution, which for Philips scanners refers to the sampling frequency of the CT detectors. For each scan, volumetric images were reconstructed using a conventional FBP algorithm and the iDose^4^ algorithm with 3 mm slice thickness setting, as well as three different reconstruction filters: A (smooth), B (standard), and C (sharp). In order to analyze the effect of pixel dimension on the calculated NPS (Eqn. 3), images were reconstructed using two fields of view (FOV): 350 mm and 500 mm. Using a 512 x 512 data matrix, this resulted in pixel sizes of 0.6836 mm x 0.6836 mm and 0.9766 mm x 0.9766 mm, respectively.

**C.4 iDose^4^ Reconstruction Algorithm**

The iDose^4^ algorithm is a 4^th^ generation reconstruction technique that was commercially released by Philips Health System (Cleveland, OH, USA). It provides improvements in image quality and radiation dose reduction through an iterative process. An adaptive linear filter is used on noisy projections in the projection domain followed by quantum noise reduction in the image domain via noise models, which provide better reduction of streak artifacts and quantum noise when compared with FBP techniques, enabling visualization of underlying anatomical information. The extent to which quantum mottle noise is reduced in an image can be controlled using iDose^4^ reconstruction levels (1-6), which corresponds to a noise reduction range from approximately 11% to 55% when compared with FBP reconstruction for the same image. By utilizing edge-preserving noise reduction techniques, the iDose^4^ reconstruction algorithm can preserve spatial resolution while correcting bias artifacts and maintaining noise power spectrum constancy. More details about iDose^4^ reconstruction algorithm can be found in the technical paper from Philips.[^31^](#_ENREF_30)

**C.5 ROI Selection**

Methodical selection of local image ROIs must be performed prior to NPS calculation. Five different square ROIs with side sizes of 32, 48, 64, 96, and 128 pixels were considered in this study for the following two reasons. First, for a 512 x 512 pixel CT image that covers a reconstruction FOV of 350 mm, the 150 mm diameter uniformity module of the Catphan® phantom will occupy approximately 220 pixels in diameter; therefore a 128-pixel ROI was used as the maximum allowable size for this study. Secondly, the smallest ROI size available for calculation is determined by the radial bin size. The Fourier transform frequency axis *f* in Equation 3 is inversely proportional to both the ROI size and the reconstructed pixel size. Given the radial bin size *r_bin_* and minimum image pixel dimension *Δp*, the smallest ROI size *S_min_* available is therefore determined by the following equation:

$S_{\min}=\left(2\times\Delta p\times r_{bin}\right)^{-1}$ (7)

This equation guarantees that each radial bin will contain a sufficient number of values for averaging. The factor of two arises from the fact that this NPS calculation method includes linear extrapolation to determine the zero frequency value, thus the first radial bin is automatically determined. For a radial bin size of 0.025 mm^-1^ and a pixel dimension of 0.6836 mm, ROI sizes smaller than 30 pixels were too small to obtain adequate radial sampling for the binning process described in Section II.A. ROI sizes with powers of two were preferred to enable faster computation of the DFT.

The ROIs were grouped both by size and by radial distance from the center of the scanned phantom to the center of the selected ROI for analysis, as with other studies utilizing cylindrical phantoms.^20-21^ To determine the center of the imaged phantom, a thresholding technique was first used to determine the real phantom region, and then the centroid of this region was calculated and used as the phantom center. Due to the reconstructed image size of the uniformity module (approximately 220 pixels in diameter), the only possible location for 128-pixel ROI was in the center (i.e. a 0 mm radius). Similarly, radii up to 56 mm were possible for 32-pixel ROI. In this study, 30 was the maximum number of 32-pixel ROIs per CT slice selected in order to guarantee that the overlap between any two adjacent ROIs was less than 50%.[^21^](#_ENREF_21) Based on visual inspection it was determined that only 16 CT slices exclusively contained the uniformity module; the aforementioned ROI selection process was performed on each of these 16 CT slices.

**III. RESULTS**

**A. Computer Simulated Data Results**

As described in Section II.C.2, the reference 1D NPS was used to evaluate the accuracy of NPS calculations with varied parameters and background removal methods. Figure 2 shows the reference 2D (Figure 2a) and corresponding 1D NPS (Figure 2b). As can be seen, the 1D NPS increases linearly in the low spatial frequency range (due to the ramp filter) followed by the high-frequency roll-off (due to the low-pass smoothing filter), resembling that reported by other published works for CT.^4, 20-21, 30^

Figure 3 displays the comparison of the NPS for four background subtraction methods and two ROI sizes for the computer simulated data, with no window function applied. While there is little dependence of the NPS on background removal method for the 128-pixel ROI, as seen in Figure 3a, the calculated NPS is dependent on the background removal method for the smallest 32-pixel ROI, particularly at low spatial frequencies. For the 32-pixel ROI, the image subtraction method most closely approximates the ground truth (see Figure 3b). While both RSS methods underestimate the low spatial frequency components, the PCA method overestimates the low frequency components. When analyzing the effect of background removal alone, the image subtraction method for background removal is the most effective, and produces similar results no matter which ROI size was used.

As with background removal method, the NPS for the simulated ROIs also depended on the window function applied to the data, as shown in Figure 4. For comparing the NPS for different window functions, all 1D results were normalized to their maximum value due to the bias introduced from the window functions. For the 128-pixel ROI the NPS is insensitive to the applied window function, but this dependence increases as the ROI size decreases. For the smallest ROI size (32 pixels), the Hann, Hamming, and Blackman-Harris window functions performed similarly well, while the flat top window function displayed the worst calculation accuracy.

Figure 5 and Table 1 demonstrate the dependence of NPS calculation accuracy on the number of ROIs used for calculation. The calculation error is defined as the average percent amplitude difference of all radial NPS data points compared to that of the reference radial NPS data points. Calculation error decreases exponentially as the ROI number increases. As shown in Figure 5a, no matter which background removal method was used, the calculation error is less than 1% for *N_ROI_* ≥ 50 when using the 128-pixel ROIs. However, when using the 32-pixel ROI, the error levels converge at different values for the different background removal methods, as seen in Figure 5b. When using all 300 ROIs, the achievable NPS calculation error levels are about 1.6%, 7.1%, 1.9%, and 4.7% for image subtraction, PCA, RSS 1^st^ order, and RSS 2^nd^ order, respectively. For the maximum number of ROIs used in this study, the image subtraction and the RSS 1^st^ order methods perform similarly, and can achieve a NPS calculation error of less than 5% when using a small number of ROIs (23 and 32, respectively). Overall, NPS calculation errors decrease as ROI size increases. A quantitative description of NPS calculation errors for two different ROI numbers, 50 and 300, is presented in Table 1 as a sample of the full results.

**B. Catphan® Phantom Results**

**B.1 NPS dependence on ROI size & location**

Figure 6 illustrates the dependence of the calculated NPS on ROI radius and image reconstruction algorithm for the 32-pixel ROI, compared to the reference NPS for the 128-pixel ROI. The calculated NPS magnitude (i.e. noise variance) for the 32-pixel ROI increases as the ROIs are closer to the center of the image due to the attenuation properties of photons. This verified the trend reported in a previous study,[^20^](#_ENREF_20) and demonstrates the importance of utilizing smaller ROIs, which contain more information about the spatial variation of noise. Statistical fluctuations in the calculated NPS decrease as the ROI radius increases, since more ROIs are available for calculation. For example, only 1 ROI per slice can be used for the 32-pixel ROI when the radius is 0 mm. However, when the radius is increased and less than 50% overlap between adjacent ROIs is allowed, a maximum of 9, 18, and 30 ROIs can be used for radii of 15, 30, and 55 mm, respectively, for the 32-pixel ROI. This is most noticeable when comparing the NPS at 0 mm radius to that at 55 mm radius for the 32-pixel ROI. The same trends are observed for both FBP and iDose^4^ reconstructions.

**B.2 NPS dependence on background removal methods**

Figure 7 shows the NPS calculation accuracy for four different background removal methods and two image reconstruction algorithms for the smallest and largest ROI side sizes, 32 and 128 pixels, with the selected ROI radii as 30 mm and 0 mm, respectively. In these instances no window function was applied in order to analyze background removal independently. While the other three background subtraction methods perform similar to image subtraction for spatial frequencies above ~0.15 mm^-1^, image subtraction is more effective at removing the low spatial frequency background components. The other three background estimation techniques overestimate the low-frequency components of the noise when compared to the image subtraction method; this trend is observed for all tested ROI sizes and for both reconstruction algorithms. When comparing Figures 7a and 7c, it is evident that the other three methods are more effective at removing low frequency noise for larger ROI sizes; however, it becomes more difficult to estimate for smaller ROIs with these three methods. Even though the NPS amplitude decreases significantly for iDose^4^ reconstructed images, the same trends were observed, as shown in Figures 7b and 7d.

**B.3 NPS dependence on window function**

As with the simulation study, the NPS calculated from the Catphan® data was insensitive to the window function for the 128-pixel ROIs. However, the NPS did demonstrate dependence on the window function for the 32-pixel ROIs, as shown in Figure 8. This dependence differed for the various background removal methods. All results are compared to the reference NPS (i.e. 128-pixel ROI, image subtraction background removal method, and no window function applied). In all instances the flat top window function did not effectively suppress the low frequency background components when compared to the reference NPS. When using the image subtraction background removal method, the other three window functions (Hann, Hamming, and Blackman-Harris) all produce similar results to the reference NPS. This was not the case for the other three background removal methods, as all window functions overestimated the low-frequency components when compared to the reference. It is interesting to note that when the PCA background removal method is used, comparable NPS shape is obtained when using all four window functions and the Hann, Hamming, and Blackman-Harris window functions achieved average percent differences of 17.5%, 18.2%, and 18.2%, respectively, when compared to the reference.

**B.4 NPS dependence on pixel dimensions**

As the pixel dimensions are a parameter of Equation 3, it is important to mention the NPS dependence on the pixel dimensions, which is demonstrated in Figure 9. As the FOV (and therefore the pixel size) increases, the NPS maintains the same magnitude and relative shape, but the full-width-half-maximum (FWHM) is truncated. This relationship is approximately inversely proportional; for this study an increase in the FOV by a factor of 1.42 resulted in a FWHM truncation by a factor of 1.30. There were no other observed differences in the calculated NPS as a function of pixel dimension for all CT acquisition and NPS calculation parameters used in this study. Thus the pixel dimensions used to analyze the NPS will not affect the results obtained in the previous three sections. However, if it is desired to adequately compare the NPS for different pixel dimension images, an appropriate FWHM scaling must be performed. Moreover, when changing the pixel dimensions the effect of spatial resolution on image quality must also be considered.

**IV. DISCUSSION**

Although NPS calculation is not a new topic in medical imaging, to the best of our knowledge, the study presented here is the first to evaluate the combined effects of varied image background removal methods with both ROI size and window function application on the local NPS calculation for CT images, reconstructed either for the conventional FBP algorithm or an iterative reconstruction algorithm. This study analyzed local NPS dependence on calculation parameters both for a simulation study and a symmetric uniformity phantom. Since the noise in CT images violates the wide-sense stationary condition, a strict interpretation of the NPS results presented in this study as the full description of image noise is forbidden. The focus of this work was on the analysis and observations of the calculation results themselves, rather than their interpretation. This study can also serve as a guide for researchers getting started in estimating the local NPS for CT.

While the 128-pixel ROI is most commonly used for NPS analysis in CT, the benefit of using a smaller, more locally defined NPS for clinically realistic data is threefold. First, the NPS varies spatially, and this spatial variation cannot be visualized as well for larger ROI sizes, since local noise properties are averaged together, with some loss of information. This point is most evident in Figure 6, as several different NPS for smaller-sized ROIs could be calculated within a single 128-pixel ROI. Additionally, it is assumed that a smaller noise sample will more closely approximate wide-sense stationary noise. Finally, considering the real size of most human anatomical structures, the smaller ROI size is more suitable for assessing the noise properties of organs and their surrounding regions. Moreover, for the Catphan® phantom the lost spatial frequency resolution that resulted from using a smaller ROI size was generally compensated for by the additional number of ROIs gained, resulting in a comparably smooth NPS. Exceptions to this observation occurred for small ROIs placed near the center of the phantom, that is, the 32-pixel ROI at 0 mm radius. In any instance of image noise assessment, the ROI size used for NPS calculation should be guided by a given task as well as limitations in the acquired data (e.g. number of image slices, size of images, size of object, desired task for image, etc). For clinical data in radiation oncology with potentially small objects, it is suggested that smaller ROI sizes are used for NPS calculation. For a given radial bin size and reconstructed image pixel size, Equation 7 can be used to determine the smallest ROI size which guarantees adequate radial sampling of the NPS, which in this instance was the 32-pixel ROI.

While smaller ROI sizes are deemed more appropriate for clinical tasks, the local NPS is more dependent on the background removal method used for calculation when smaller ROI sizes are used, as shown from both the simulation and Catphan® studies. For all clinically acquired images utilized in this study, the image subtraction method of background removal was the most effective at removing low-frequency noise correlations. This is understandable since the subtraction process of two separate images of the same object should directly remove any non-stochastic features in the image. This method therefore also removes object dependence, allowing for analysis of ROIs containing multiple structures. Many previous NPS calculation methodologies use an estimation type of background removal method, and some subsequently fit the NPS data to a polynomial function. In either case it is strongly recommended that the final result be compared to image subtraction for NPS calculation accuracy, especially in the low spatial frequency domain. The choice of background removal method used for NPS calculation should be guided by the number of sequential image sets available. Typically, the acquisition of two identical images of patients is not feasible in a clinical setting, so it is desirable to find a method of background estimation which produces similar results as that for image subtraction but only requires one CT acquisition.

The local NPS is also more dependent on the applied window function when smaller ROI sizes are used. The Hann, Hamming, and Blackman-Harris window functions all produced similar results to the reference NPS when image subtraction was used, and can improve results for the other background removal methods. In this study, the PCA method with application of a Hann window function most closely approximated image subtraction, and illustrates the potential for a combination of background removal method and window function which achieves similar accuracy to the image subtraction method. Yet, the deficiency of these methods in the low-frequency domain still remains. Therefore, a further in-depth analysis of this variable in NPS calculations will be investigated for future research, with the goal of finding an acceptable and reasonable alternative to image subtraction for patient data analysis.

When comparing the results of the simulation study to that of the Catphan® study, it can be seen that the same trends for background removal are not observed. For example, while both the image subtraction and RSS first-order methods produce similar NPS results for the simulation study, for the Catphan® study the image subtraction method removes the low-frequency background components much more effectively when compared to the RSS first-order method. This is due to the fact that the simulated ROIs with a predetermined NPS cannot completely model the properties of clinically acquired image data. A different background trend needs to be determined to more closely approximate real clinical images. Despite this limitation, the use of a pre-determined NPS as the ground truth in the simulation study allowed for absolute analysis of the calculation parameters, in addition to the relative NPS comparison using the Catphan® phantom.

It was observed in this study is that the same trends for ROI size, background removal method, and window function were observed for both FBP and iDose^4^. However, this does not necessarily indicate a similarity of noise properties between FBP and iDose^4^, as the NPS is not the full description of the noise. It is expected that the off-diagonal elements of the DFT of the covariance matrix will vary with reconstruction algorithm, which would be an interesting area of future research. Moreover, since each iterative algorithm uses a unique noise reduction strategy, the results found in this study may not hold true when other iterative algorithms are considered. The NPS of each iterative algorithm should therefore be assessed separately.

While this study can be applied generally to clinical CT scanners, the intended long-term aim for this study specifically involves CT scanners in radiation therapy. The NPS is often used individually to characterize imaging system noise, but it can also be included within the context of a task-based image quality assessment framework. In this paradigm the NPS, MTF, and other metrics are used to calculate scalar figures of merit (FOM) such as the signal-to-noise ratio (SNR) for various model observers (e.g. Ideal Bayesian, Hotelling). These FOM are then used to objectively and quantitatively assess the performance of imaging systems for specific tasks. The majority of imaging tasks are diagnostic (i.e. detecting the presence of disease), but in radiation therapy the tasks are not diagnostic in nature and include, among others, image segmentation and dose calculation. In radiation therapy, CT simulation image quality should be quantified and optimized based on its intended tasks. The purpose of this preliminary study is to provide an analysis of the NPS for CT, which will inform and guide the simulation CT image optimization process. Future work will include a comprehensive study of the effect of noise at different spatial frequencies, as well as the effect of off-diagonal elements outside the NPS, on specific radiation therapy tasks such as image segmentation. This will enable optimization of all available imaging parameters for specific radiotherapy tasks.

**V. CONCLUSION**

The local NPS varies depending on calculation parameters such as the ROI size, background removal method, and window function, particularly at low spatial frequencies. Image noise should be analyzed on a local level by calculating the NPS for small ROI sizes, in order to capture the spatial variation of noise properties and more closely approximate the condition of wide-sense stationary noise. A minimum ROI size is recommended based on the chosen radial bin size and image pixel dimensions. As the ROI size decreases the NPS becomes more dependent on the choice of background removal method and window function. The image subtraction method is most accurate, but other methods can achieve similar accuracy if certain window functions are applied. All dependencies should be analyzed and taken into account when considering the interpretation of the NPS for task-based image quality assessment.

**REFERENCES**

^1^ K. Faulkner, B.M. Moores, "Analysis of x-ray computed tomography images using the noise power spectrum and autocorrelation function," Phys Med Biol **29**, 1343-1352 (1984).

^2^ J.T. Dobbins, 3rd, D.L. Ergun, L. Rutz, D.A. Hinshaw, H. Blume, D.C. Clark, "DQE(f) of four generations of computed radiography acquisition devices," Med Phys **22**, 1581-1593 (1995).

^3^ S.J. Riederer, N.J. Pelc, D.A. Chesler, "The noise power spectrum in computed X-ray tomography," Physics in Medicine and Biology **23**, 446 (1978).

^4^ K.L. Boedeker, V.N. Cooper, M.F. McNitt-Gray, "Application of the noise power spectrum in modern diagnostic MDCT: part I. Measurement of noise power spectra and noise equivalent quanta," Physics in Medicine and Biology **52**, 4027 (2007).

^5^ K.L. Boedeker, M.F. McNitt-Gray, "Application of the noise power spectrum in modern diagnostic MDCT: part II. Noise power spectra and signal to noise," Physics in Medicine and Biology **52**, 4047 (2007).

^6^ R.F. Wagner, D.G. Brown, "Unified SNR analysis of medical imaging systems," Physics in Medicine and Biology **30**, 489 (1985).

^7^ R.F. Wagner, D.G. Brown, "Overview of a Unified SNR Analysis of Medical Imaging Systems," IEEE Trans Med Imaging **1**, 210-213 (1982).

^8^ R.F. Wagner, D.G. Brown, M.S. Pastel, "Application of information theory to the assessment of computed tomography," Med Phys **6**, 83-94 (1979).

^9^ R.F. Wagner, C.E. Metz, G. Campbell, "Assessment of medical imaging systems and computer aids: a tutorial review," Acad Radiol **14**, 723-748 (2007).

^10^ E. Samei, M.J. Flynn, "An experimental comparison of detector performance for computed radiography systems," Medical Physics **29**, 447-459 (2002).

^11^ M.B. Williams, P.A. Mangiafico, P.U. Simoni, "Noise power spectra of images from digital mammography detectors," Med Phys **26**, 1279-1293 (1999).

^12^ R. Garcia-Molla, R. Linares, R. Ayala, "Study of DQE dependence with beam quality on GE essential mammography flat panel," Journal of applied clinical medical physics / American College of Medical Physics **12**, 3176 (2011).

^13^ D.S. Brettle, A. Workman, R.P. Ellwood, J.H. Launders, K. Horner, R.M. Davies, "The imaging performance of a storage phosphor system for dental radiography," The British journal of radiology **69**, 256-261 (1996).

^14^ D.A. Jaffray, J.H. Siewerdsen, "Cone-beam computed tomography with a flat-panel imager: Initial performance characterization," Medical Physics **27**, 1311-1323 (2000).

^15^ J.H. Siewerdsen, L.E. Antonuk, Y. El-Mohri, J. Yorkston, W. Huang, I.A. Cunningham, "Signal, noise power spectrum, and detective quantum efficiency of indirect-detection flat-panel imagers for diagnostic radiology," Medical Physics **25**, 614-628 (1998).

^16^ C. Ghetti, F. Palleri, G. Serreli, O. Ortenzia, L. Ruffini, "Physical characterization of a new CT iterative reconstruction method operating in sinogram space," Journal of applied clinical medical physics / American College of Medical Physics **14**, 4347 (2013).

^17^ K. Matsubara, H. Koshida, K. Sakuta, T. Takata, J. Horii, H. Iida, K. Koshida, K. Ichikawa, O. Matsui, "Radiation dose and physical image quality in 128-section dual-source computed tomographic coronary angiography: a phantom study," Journal of applied clinical medical physics / American College of Medical Physics **13**, 3959 (2012).

^18^ H.H. Barrett, K.J. Myers, "Foundations of image science," Foundations of Image Science, by Harrison H. Barrett, Kyle J. Myers, pp. 1584. ISBN 0-471-15300-1. Wiley-VCH, October 2003. **1**2003).

^19^ S.A.H. C. C. Brunner, S. F. Abboud, C. Hoeschen, and I. S., Kyprianou, "Noise characterization of computed tomography using the covariance matrix," Proc. SPIE **7622**2010).

^20^ A.R. Pineda, D.J. Tward, A. Gonzalez, J.H. Siewerdsen, "Beyond noise power in 3D computed tomography: the local NPS and off-diagonal elements of the Fourier domain covariance matrix," Medical physics **39**, 3240-3252 (2012).

^21^ S.N. Friedman, G.S.K. Fung, J.H. Siewerdsen, B.M.W. Tsui, "A simple approach to measure computed tomography (CT) modulation transfer function (MTF) and noise-power spectrum (NPS) using the American College of Radiology (ACR) accreditation phantom," Medical physics **40**, 051907 (2013).

^22^ F.A. Miéville, F. Gudinchet, F. Brunelle, F.O. Bochud, F.R. Verdun, "Iterative reconstruction methods in two different MDCT scanners: Physical metrics and 4-alternative forced-choice detectability experiments – A phantom approach," Physica Medica **29**, 99-110 (2013).

^23^ B. Chen, O. Christianson, J.M. Wilson, E. Samei, "Assessment of volumetric noise and resolution performance for linear and nonlinear CT reconstruction methods," Medical physics **41**, 071909 (2014).

^24^ J.H. Siewerdsen, I.A. Cunningham, D.A. Jaffray, "A framework for noise-power spectrum analysis of multidimensional images," Medical Physics **29**, 2655-2671 (2002).

^25^ J.Y. Vaishnav, W.C. Jung, L.M. Popescu, R. Zeng, K.J. Myers, "Objective assessment of image quality and dose reduction in CT iterative reconstruction," Medical physics **41**, 071904 (2014).

^26^ Z. Zhou, F. Gao, H. Zhao, L. Zhang, "Techniques to improve the accuracy of noise power spectrum measurements in digital x-ray imaging based on background trends removal," Medical physics **38**, 1600-1610 (2011).

^27^ A. Nurunnabi, D. Belton, G. West, "Robust statistical approaches for local planar surface fitting in 3D laser scanning data," Isprs Journal of Photogrammetry and Remote Sensing **96**, 106-122 (2014).

^28^ H. Hoppe, De Rose, T., Duchamp, T., "Surface reconstruction from unorganized points," Proc. of ACM SIGGRAPH **26**, 71-78 (1992).

^29^ F.J. Harris, "On the use of windows for harmonic analysis with the discrete Fourier transform," Proceedings of the IEEE **66**, 51-83 (1978).

^30^ M.F. Kijewski, P.F. Judy, "The noise power spectrum of CT images," Physics in Medicine and Biology **32**, 565 (1987).

^31^ "iDose4 iterative reconstruction technique," in [*http://www.healthcare.philips.com/pwc_hc/main/shared/assets/documents/ct/idose_white_paper_452296267841.pdf*](http://www.healthcare.philips.com/pwc_hc/main/shared/assets/documents/ct/idose_white_paper_452296267841.pdf) (Philips Healthcare System, 2012).

| 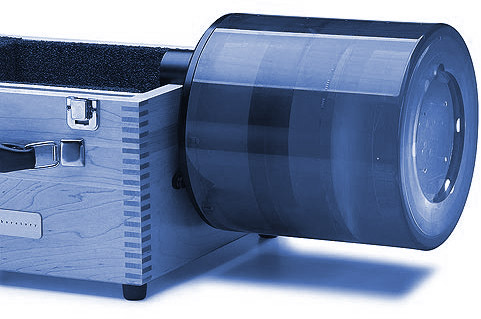 | 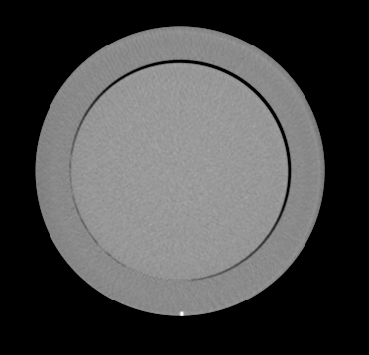 |
| --- | --- |
| (a) | (b) |
| **Fig. 1.** The Catphan® 504 phantom (a) and one CT image slice of the uniform module (b). The arrow in panel (a) indicates the uniformity module location. | |

| **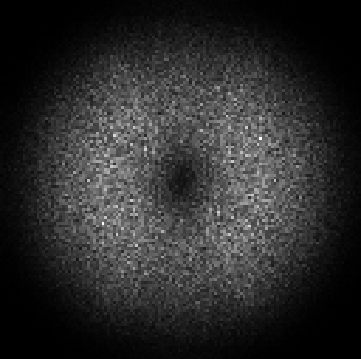** | **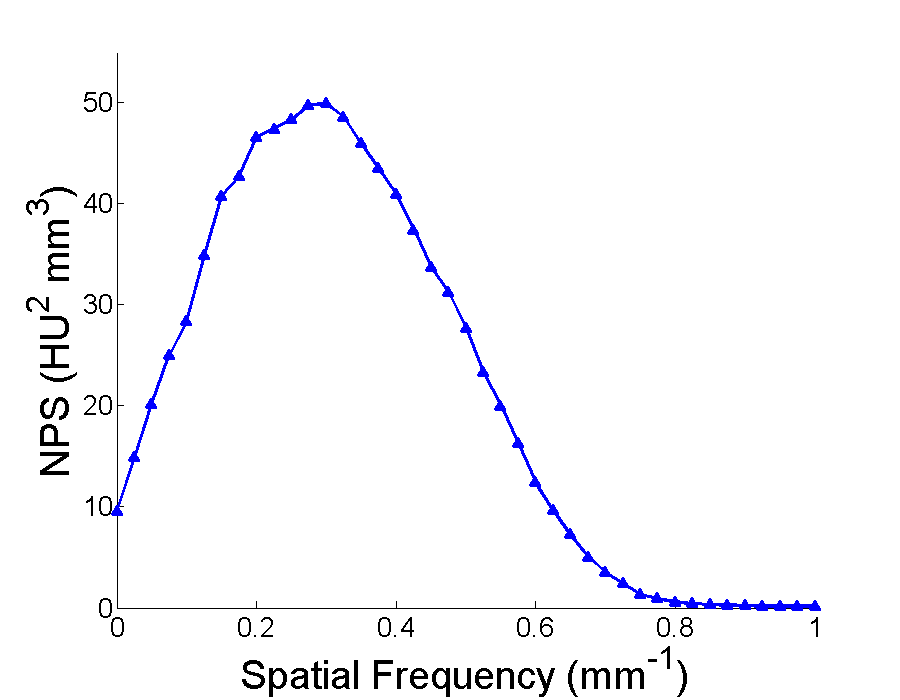** |
| --- | --- |
| (a) | (b) |
| **Fig. 2.** The reference 2D (a) and radial 1D (b) NPS calculated with a 128-pixel ROI and image subtraction as the background removal method. The ROI was selected from a CT image acquired using 120 kVp and 250 effective mAs, and reconstructed using FBP with the sharp (C) filter. | |

| 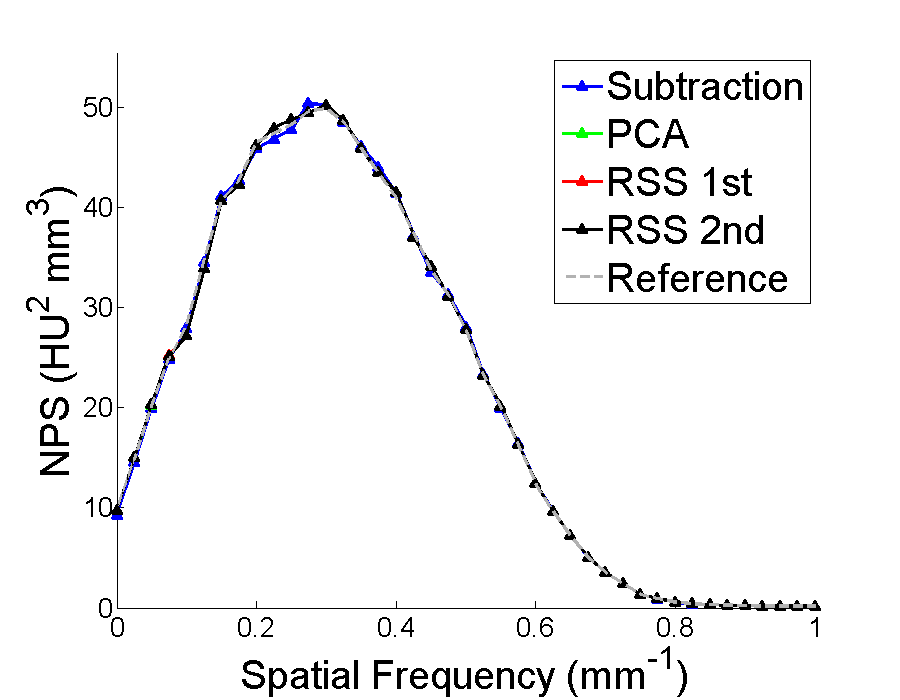 | 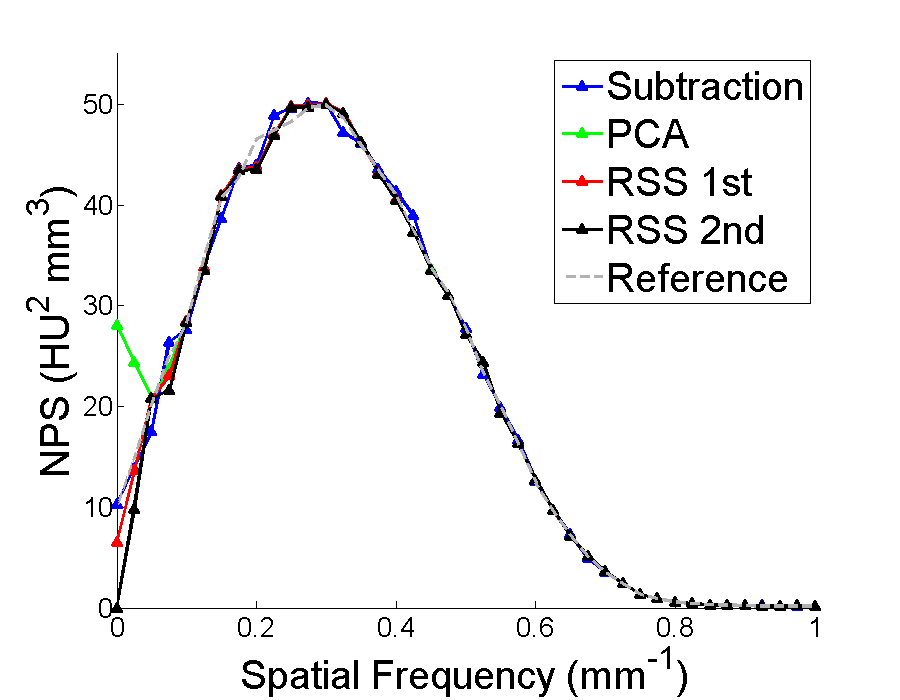 |
| --- | --- |
| (a) 128-pixel ROI | (b) 32-pixel ROI |
| **Fig. 3.** The comparison of the calculated NPS for four background subtraction methods on the computer simulated ROIs with side sizes of 128 pixels (a) and 32 pixels (b). | |

| 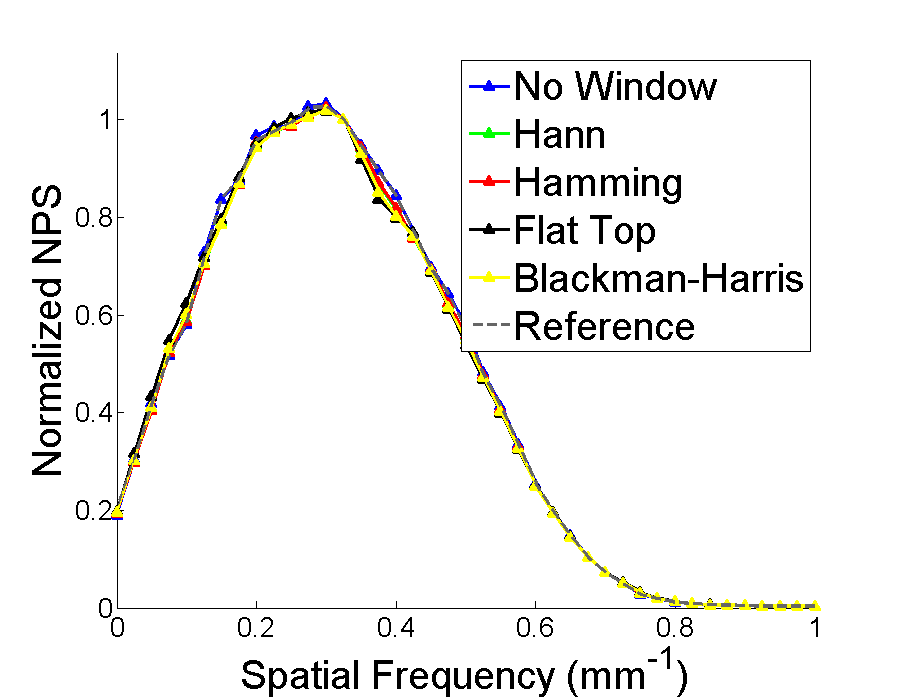 | 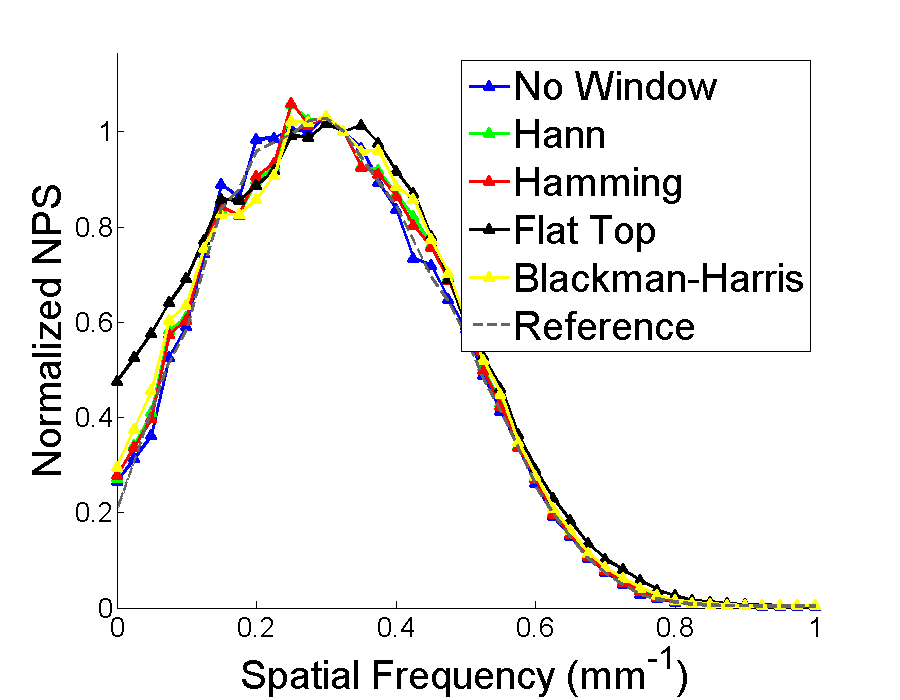 |
| --- | --- |
| (a) 128-pixel ROI | (b) 32-pixel ROI |
| **Fig. 4.** The comparison of the calculated NPS for four window functions on the computer simulated ROIs with side sizes of 128 pixels (a) and 32 pixels (b), using the image subtraction background removal method. | |

| 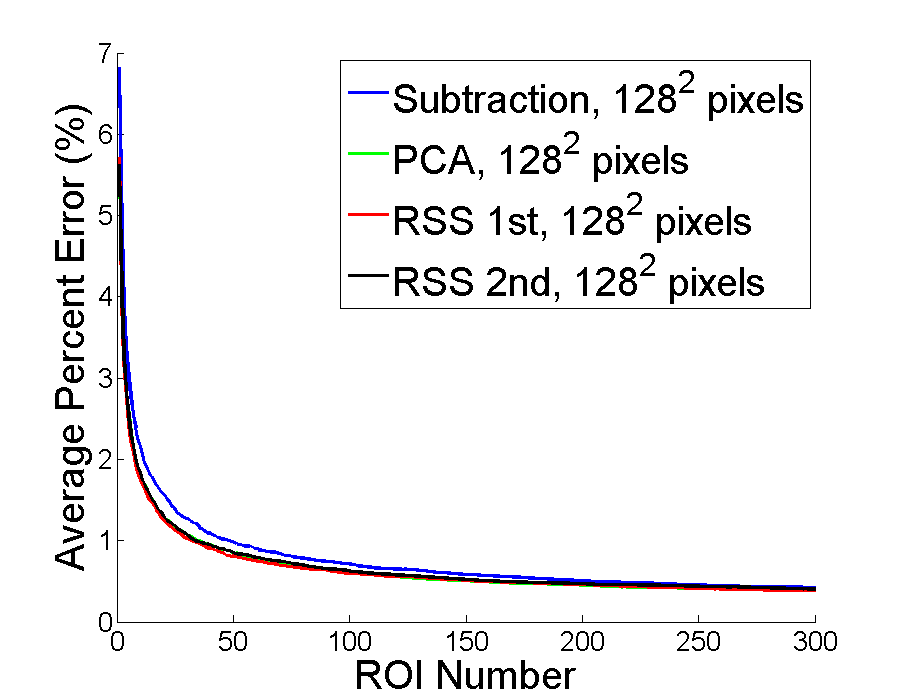 | 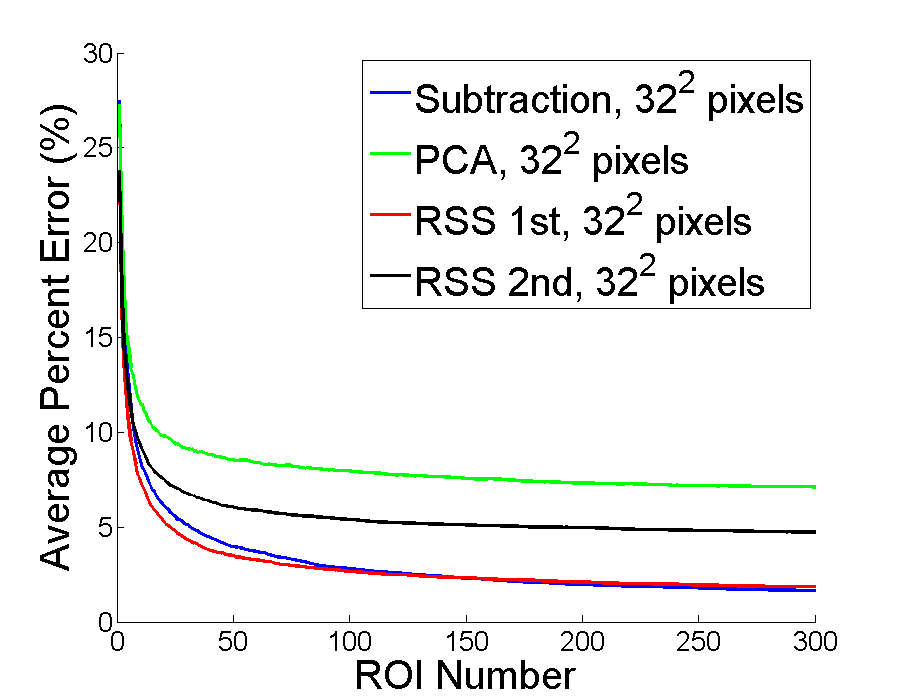 |
| --- | --- |
| (a) 128-pixel ROI | (b) 32-pixel ROI |
| **Fig. 5**. The NPS calculation error as a function of the number of averaged ROIs for various ROI sizes and background removal methods. The comparisons are on the computer simulated 128-pixel (a) and on the 32-pixel (b) ROIs. No window function was applied. | |

| 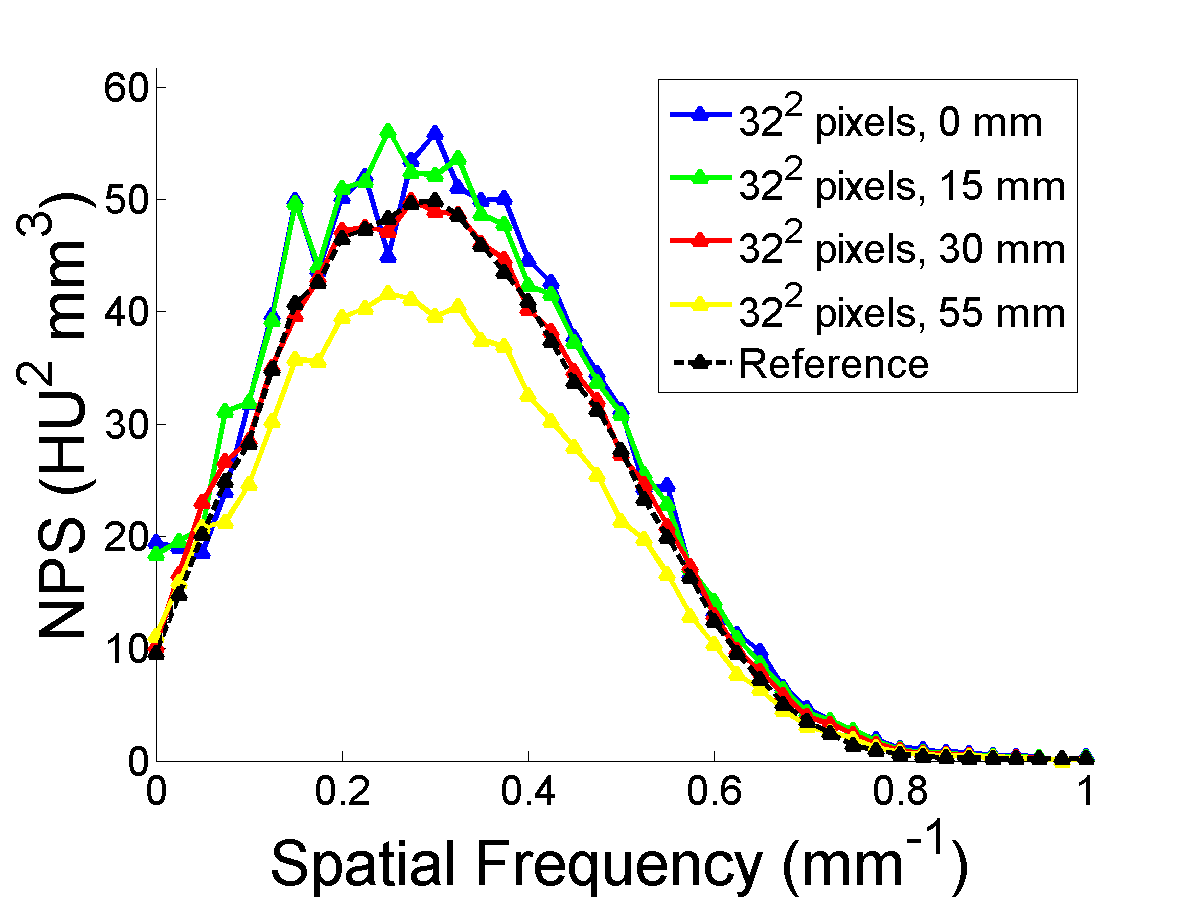 | 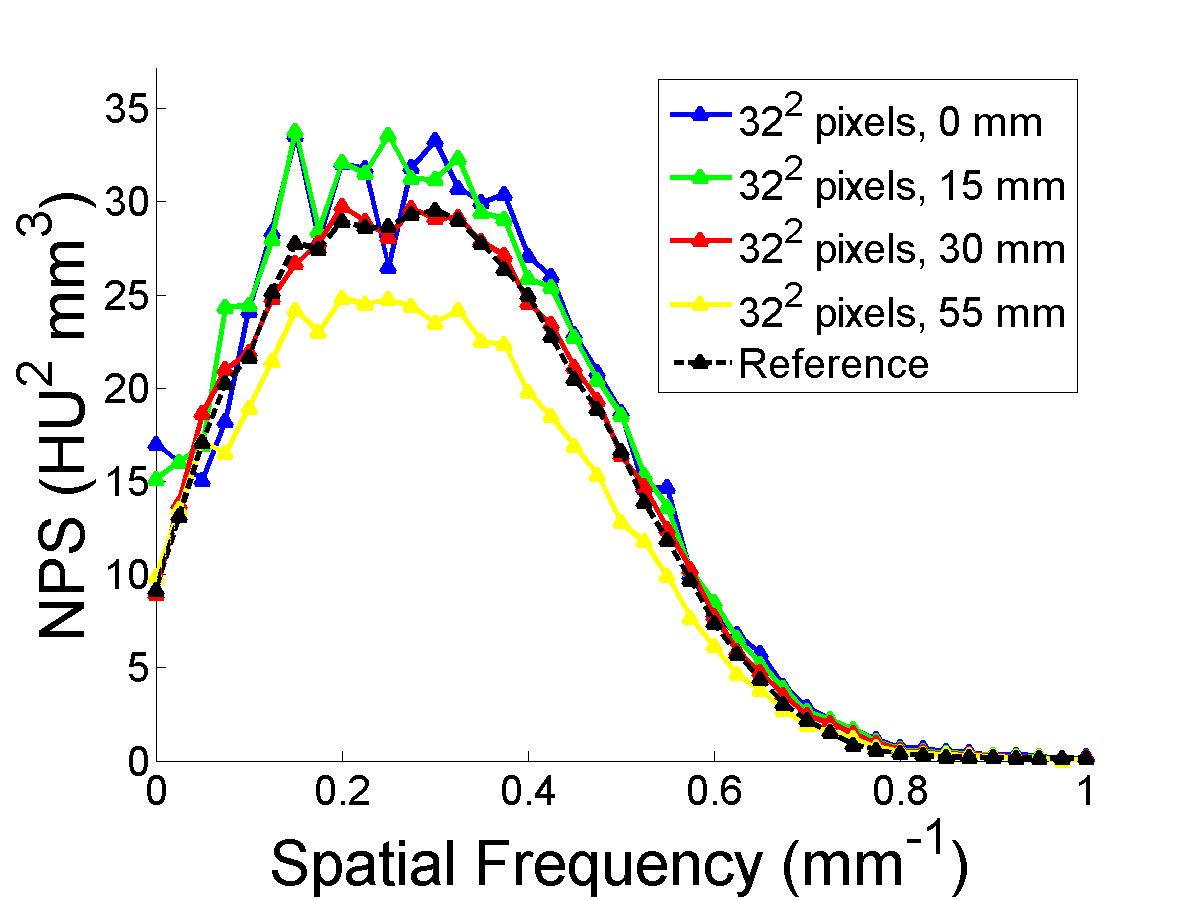 |
| --- | --- |
| (a) FBP reconstruction | (b) iDose^4^ reconstruction |
| **Fig. 6.** The comparison of NPS calculated with varied ROI radii for 32-pixel ROIs, compared to the reference NPS using the 128-pixel ROI. The ROIs were selected from images that were acquired with parameters: 120 kVp, 250 effective mAs, and sharp (C) filter. The other scanning parameters are the same as that shown in Section II.C.3. | |

| 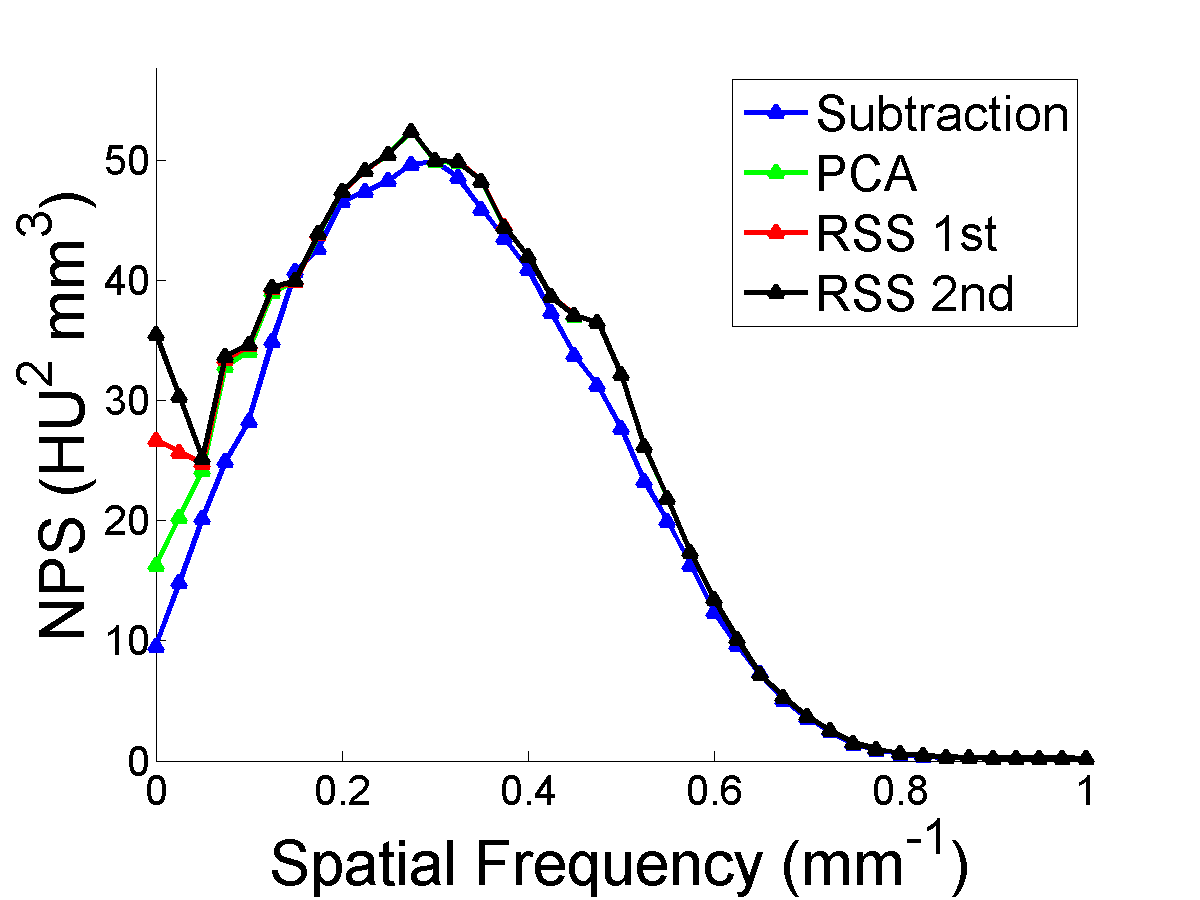 | 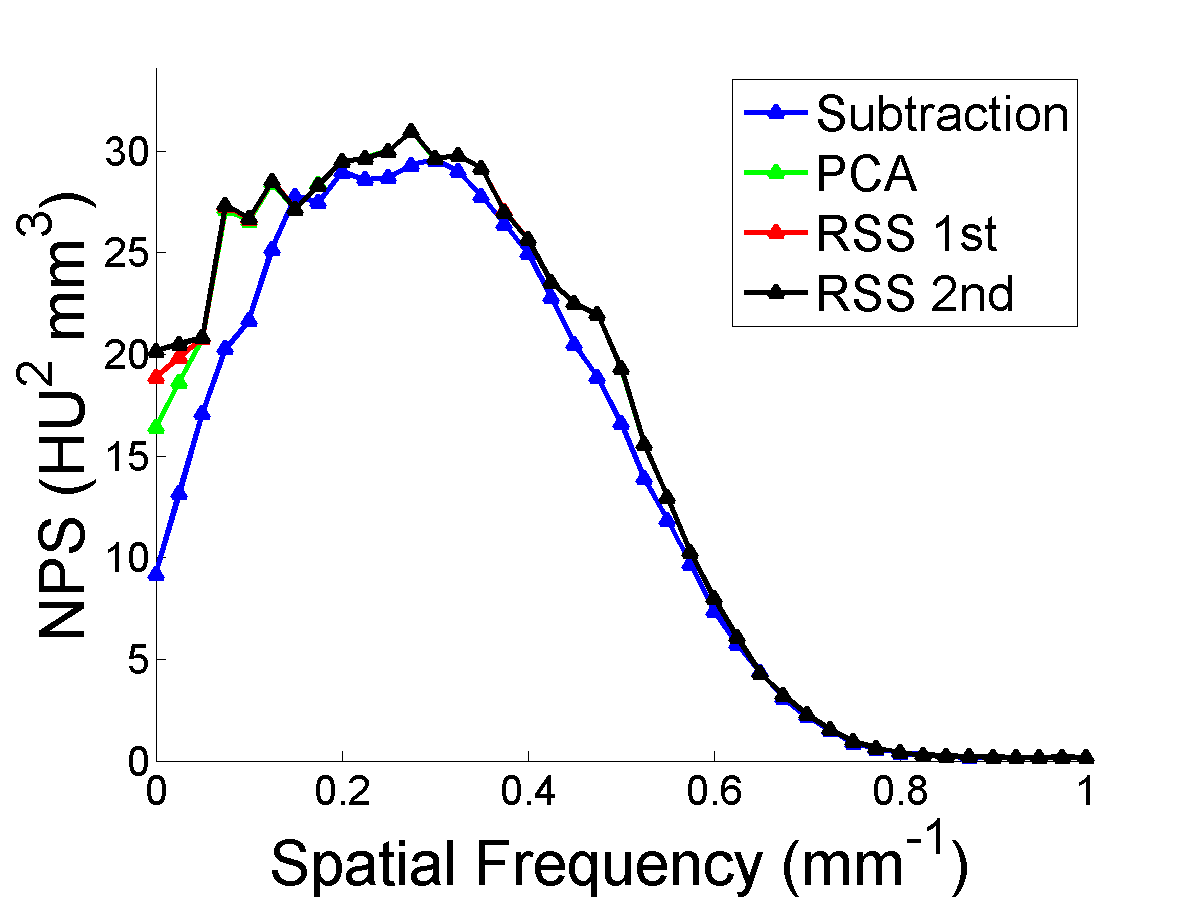 |
| --- | --- |
| (a) 128-pixel ROIs, FBP reconstruction | (b) 128-pixel ROIs, iDose^4^ reconstruction |
| 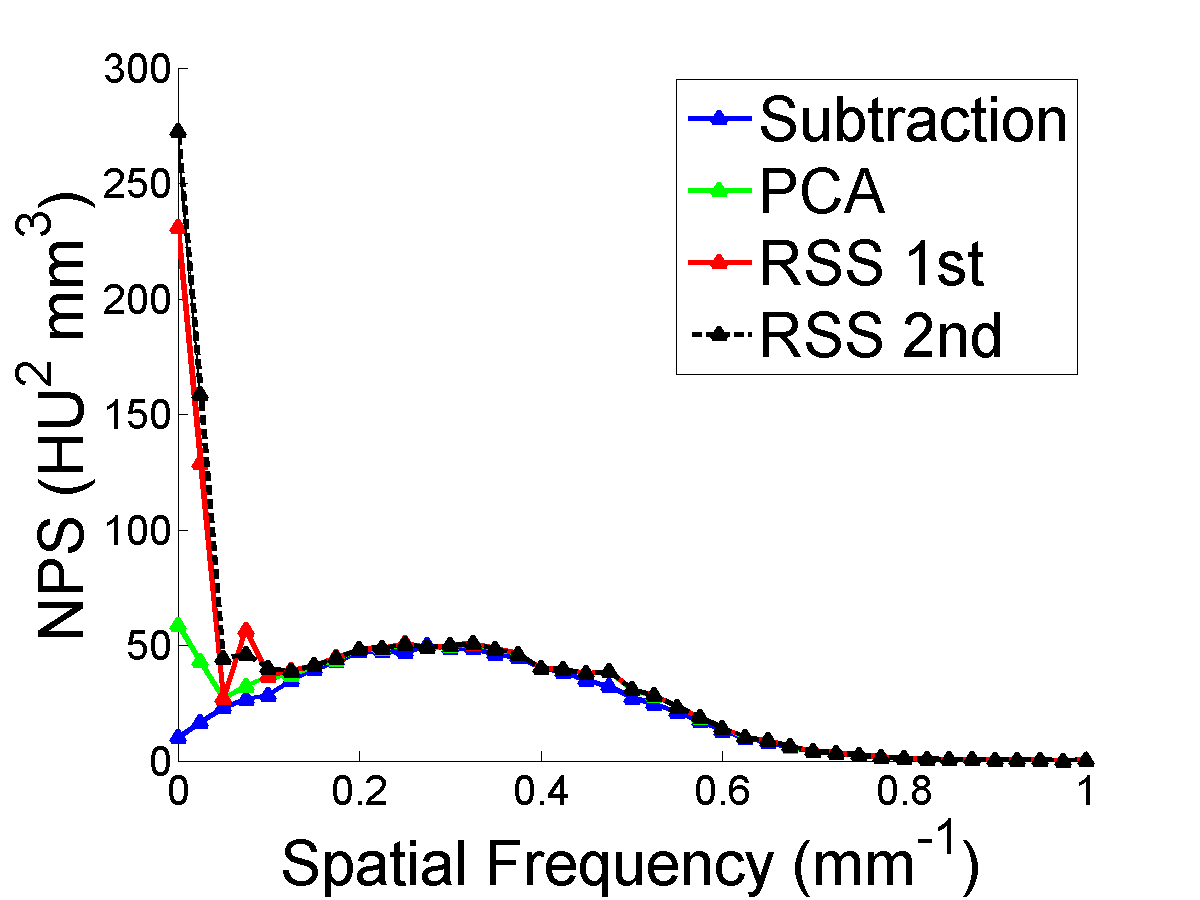 | 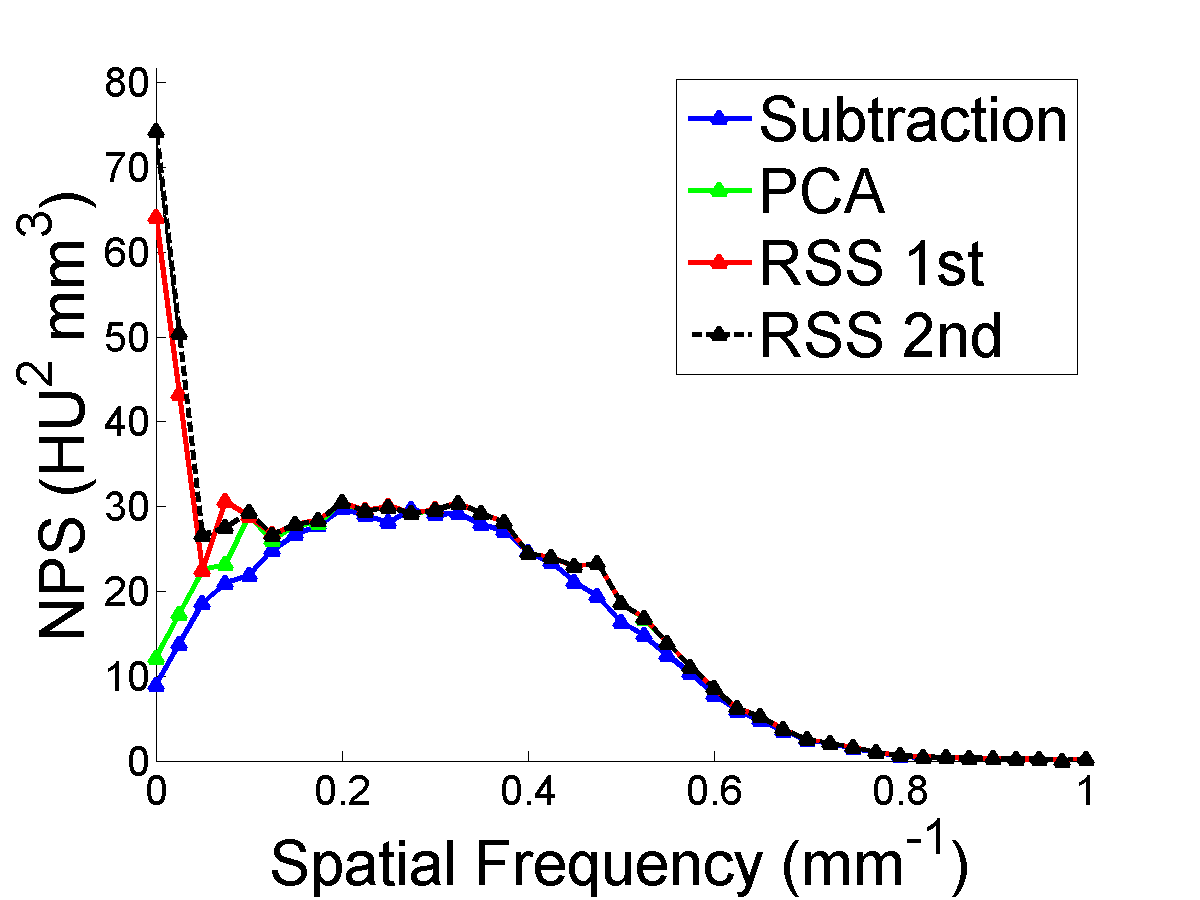 |
| (c) 32-pixel ROIs, FBP reconstruction | (d) 32-pixel ROIs, iDose^4^ reconstruction |
| **Fig. 7.** The comparison of calculated NPS using four background estimation methods, for the 128-pixel (a, b) and the 32-pixel (c, d) ROIs, using FBP (a, c) and iDose^4^ reconstruction with noise reduction level 3 (b, d) algorithms. The ROI radius for the 128-pixel and 32-pixel ROIs are 0 and 30 mm, respectively. The ROIs were selected from the image acquired with the following parameters: 120 kVp, 250 effective mAs, and a sharp (C) reconstruction filter. The other scanning parameters are the same as that explained in Section II.C.3. | |

| 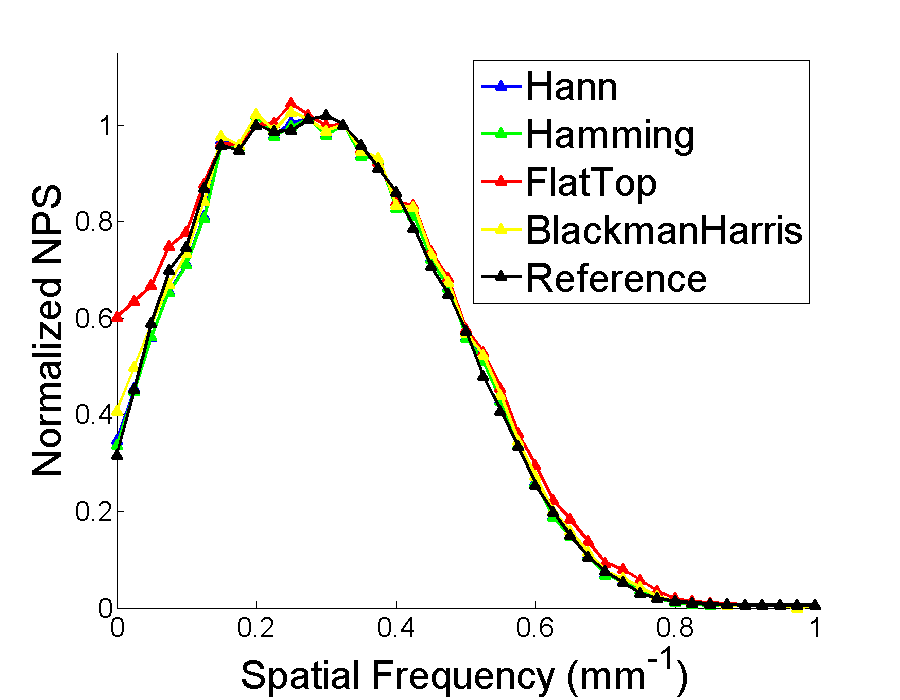 | 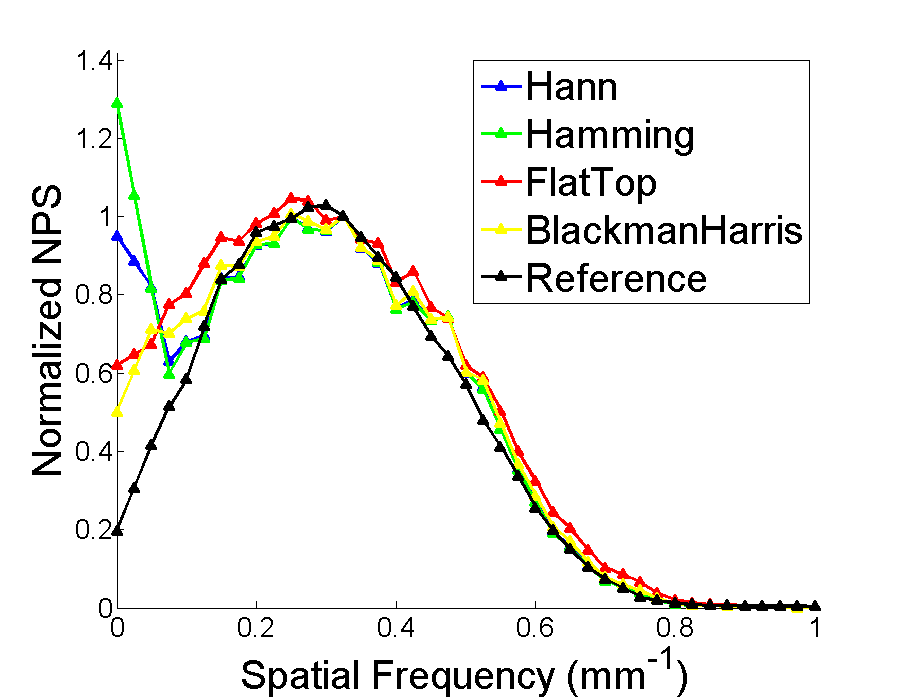 |
| --- | --- |
| (a) Image subtraction | (b) RSS 1^st^ order |
| 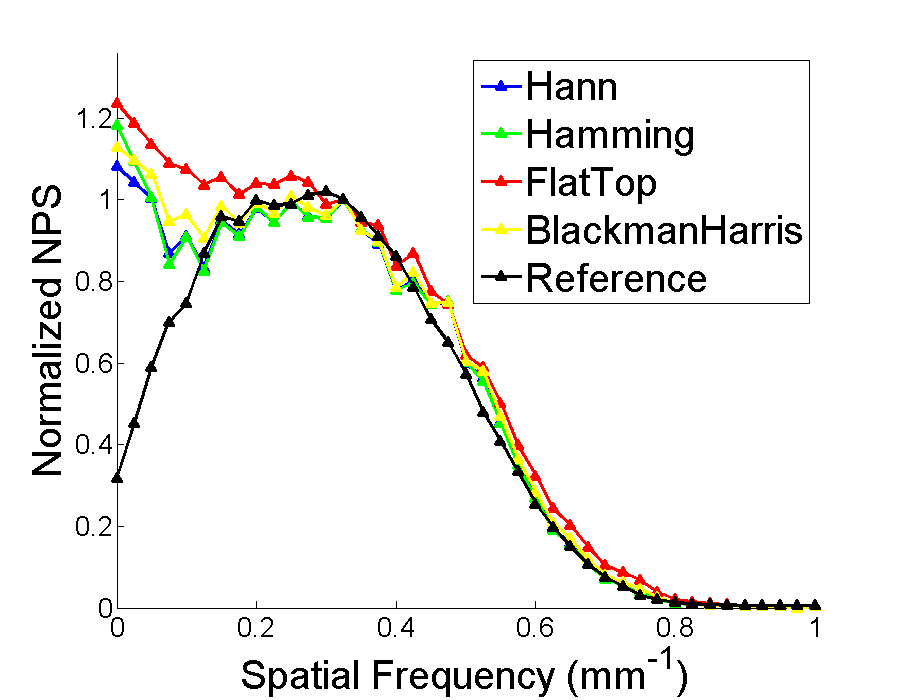 | 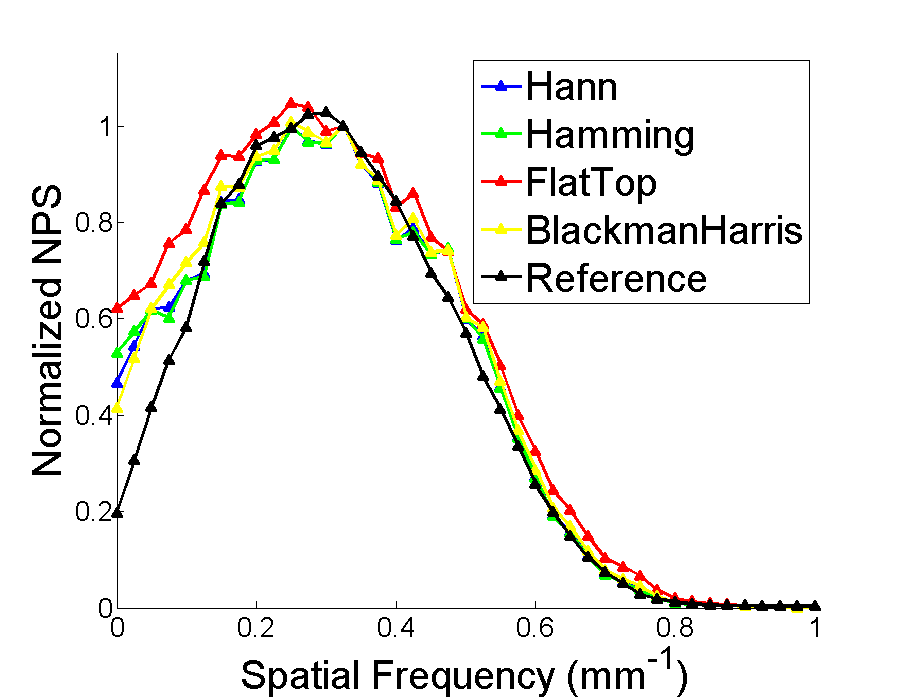 |
| (c) RSS 2^nd^ order | (d) PCA |
| **Fig. 8.** The comparison of calculated NPS using four window functions, for the 32-pixel ROIs, using the FBP reconstruction algorithm. The ROI radius for the 32-pixel ROIs is 30 mm. The ROIs were selected from the image acquired with the following parameters: 120 kVp, 250 effective mAs, and a sharp (C) reconstruction filter. The other scanning parameters are the same as that explained in Section II.C.3. | |

| 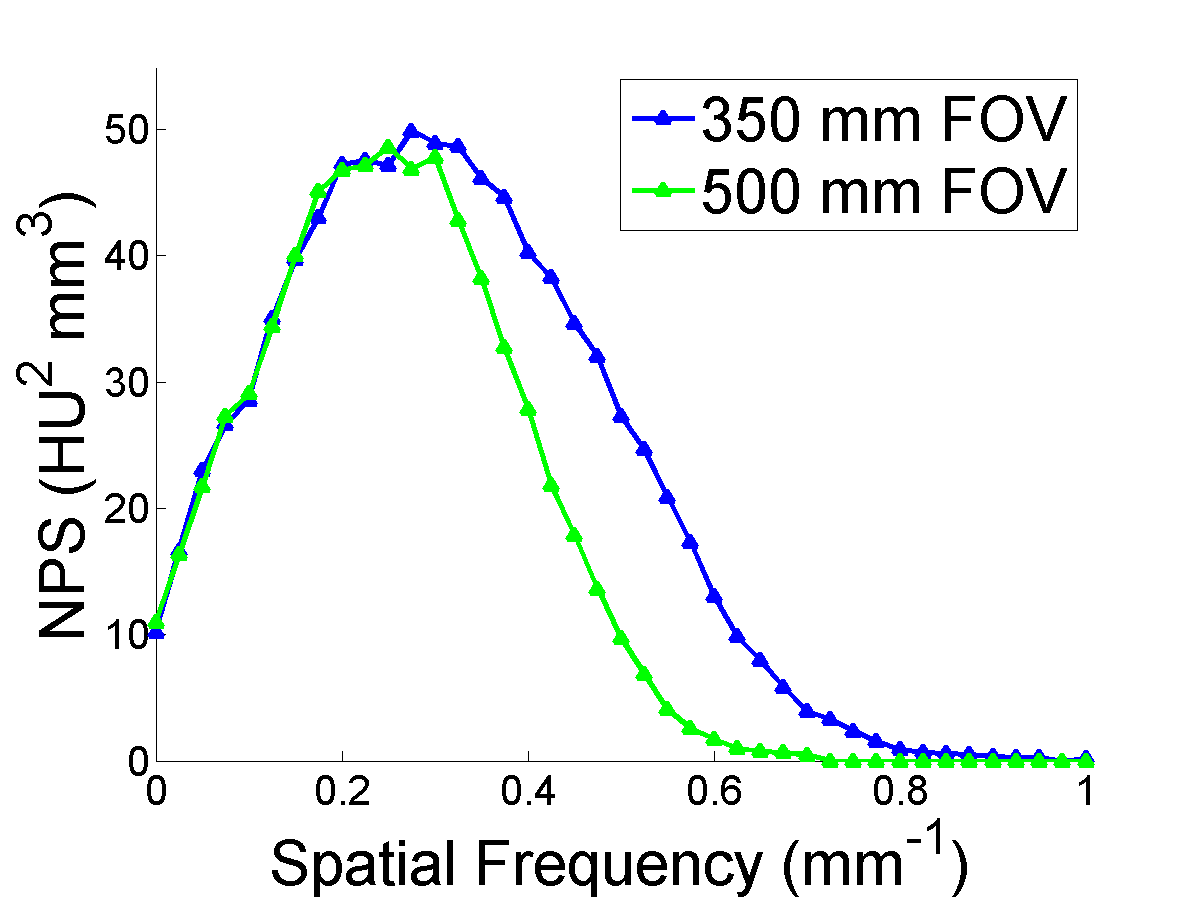 | 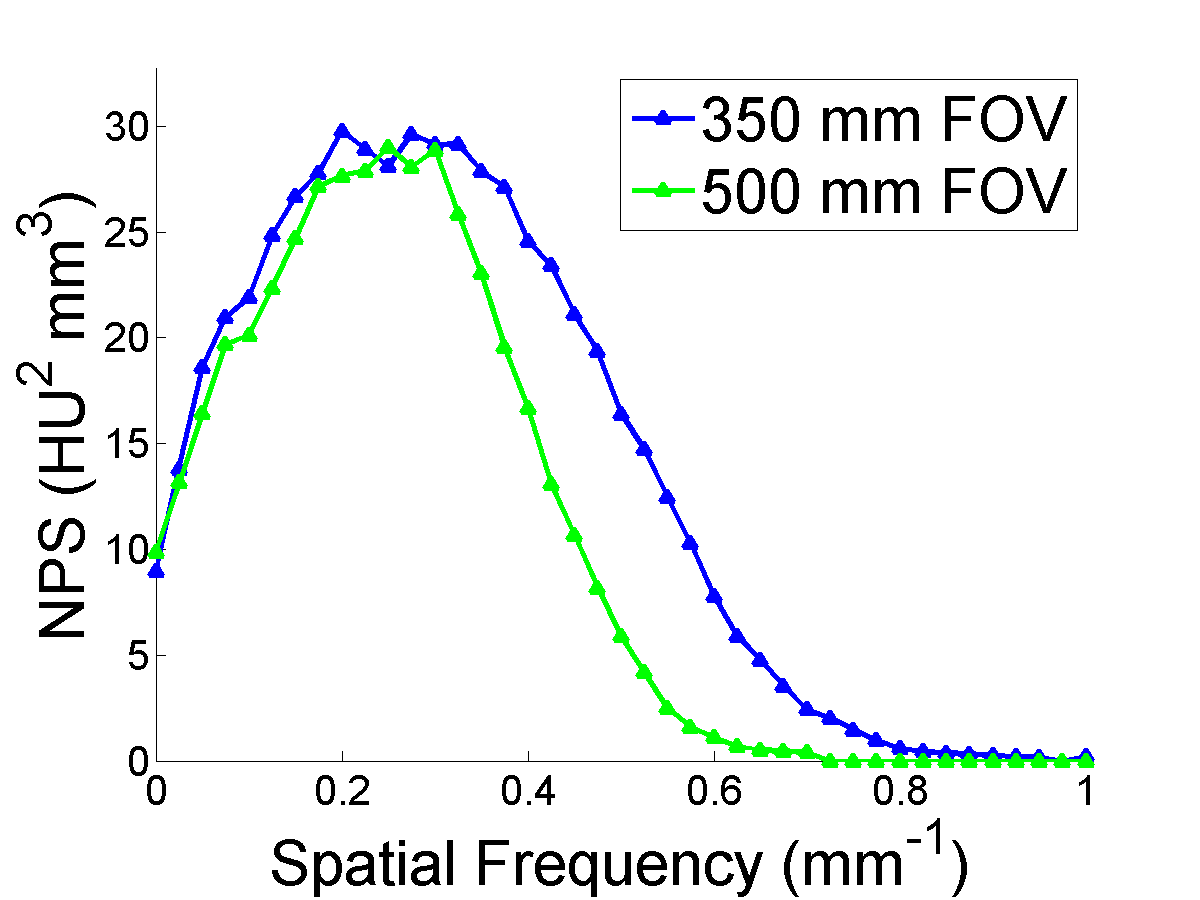 |
| --- | --- |
| (a) FBP reconstruction | (b) iDose^4^ reconstruction (level 3) |
| **Fig 9.** The comparison of calculated NPS using two pixel sizes for both the FBP and iDose^4^ (level 3) reconstruction algorithms. The NPS were calculated using a 32-pixel ROI with a radius of 30 mm. The ROIs were selected from the image acquired with the following parameters: 120 kVp, 250 effective mAs, and a sharp (C) reconstruction filter. The other scanning parameters are the same as that explained in Section II.C.3. | |

| **NPS Calculation Error (%)** | | | | | |
| --- | --- | --- | --- | --- | --- |
| **Averaged ROI Number** | **ROI Size (pixels)** | **Background removal methods** | | | |
|  |  | **Image Subtraction** | **PCA** | **RSS 1st Order** | **RSS 2nd Order** |
| **50** | 128 | 0.98 | 0.85 | 0.81 | 0.85 |
|  | 96 | 1.32 | 1.16 | 1.11 | 1.15 |
|  | 64 | 2.02 | 1.80 | 1.71 | 1.89 |
|  | 48 | 2.69 | 2.30 | 2.24 | 3.58 |
|  | 32 | 3.98 | 8.52 | 3.49 | 6.05 |
| **300** | 128 | 0.42 | 0.39 | 0.38 | 0.40 |
|  | 96 | 0.54 | 0.58 | 0.51 | 0.59 |
|  | 64 | 0.83 | 0.92 | 0.81 | 1.01 |
|  | 48 | 1.07 | 1.18 | 1.16 | 2.59 |
|  | 32 | 1.64 | 7.11 | 1.85 | 4.73 |

**Table 1.** NPS average percent error for different ROI number, ROI size, and background removal method. No window functions were applied for this data.
